# Enhanced tolerance of industrial hemp (*Cannabis sativa* L.) plants on abandoned mine land soil leads to overexpression of cannabinoids

**DOI:** 10.1371/journal.pone.0221570

**Published:** 2019-08-29

**Authors:** Rabab Husain, Hannah Weeden, Daniel Bogush, Michihito Deguchi, Mario Soliman, Shobha Potlakayala, Ramesh Katam, Stephen Goldman, Sairam Rudrabhatla

**Affiliations:** 1 Penn State Harrisburg, Middletown, PA, United States of America; 2 Department of Biological Sciences, Florida A&M University, Tallahassee, FL, United States of America; 3 Burrell College of Osteopathic Medicine, Las Cruces, NM, United States of America; 4 Department of Environmental Sciences, The University of Toledo, Toledo, OH, United States of America; The University of Newcastle, AUSTRALIA

## Abstract

Industrial activities have a detrimental impact on the environment and health when high concentrations of pollutants are released. Phytoremediation is a natural method of utilizing plants to remove contaminants from the soil. The goal of this study was to investigate the ability of *Cannabis sativa* L. to sustainably grow and remediate abandoned coal mine land soils in Pennsylvania. In this study, six different varieties of industrial hemp (Fedora 17, Felina 32, Ferimon, Futura 75, Santhica 27, and USO 31) were grown on two different contaminated soil types and two commercial soils (Miracle-Gro Potting Mix and PRO-MIX HP Mycorrhizae High Porosity Grower Mix). Plants growing in all soil types were exposed to two environmental conditions (outside and in the greenhouse). Seed germination response and plant height indicated no significant differences among all hemp varieties grown in different soils, however on an average, the height of the plants grown in the greenhouse exceeded that of the plants grown outdoors. In addition, heavy metal analysis of Arsenic, Lead, Nickel, Mercury, and Cadmium was performed. The concentration of Nickel was 2.54 times greater in the leaves of hemp grown in mine land soil outdoors when compared to greenhouse conditions. No differences were found between expression of heavy metal transporter genes. Secondary metabolite analysis of floral buds from hemp grown in mine land soil displayed a significant increase in the total Cannabidiol content (2.16%, 2.58%) when compared to Miracle-Gro control soil (1.08%, 1.6%) for outdoors and in the greenhouse, respectively. Molecular analysis using qRT-PCR indicated an 18-fold increase in the expression of the cannabidiolic acid synthase gene in plants grown on mine land soil. The data indicates a high tolerance to heavy metals as indicated from the physiological and metabolites analysis.

## Introduction

Depletion of the ozone layer and global warming are two of the biggest issues at hand due to the release of toxic pollutants into the environment [1]. Inorganic pollutants include heavy metals such as Fe, Mn, Zn, Cu, Mg, Mo, and Ni, which are necessary for plant growth but are detrimental to the environment at high concentrations in the soil. Leaching of these metals into surrounding areas through rainwater runoff poses a dangerous environmental and health risk [2]. Metals with an unknown biological purpose such as Cd, Cr, Pb, Co, Ag, Se, and Hg can also become accumulated and in high amounts be toxic [3]. Phytoremediation is a natural, cost-effective process in which plants are used to remove unsafe compounds from the soil [4], [5]. Many plants have been studied and deemed effective at removing toxins from the soil. However, only a small number of those plants have numerous beneficial applications outside of phytoremediation. Methods of extraction and disposal of these plants have not been well defined [2], [6].

Industrial hemp has been reported to be a hyperaccumulator [2], [7], [8], [9]. A previous study confirmed metal accumulation in both hemp roots and above ground tissues without noticeable changes in plant growth and morphology. However, the mechanism of accumulation remained unexplained [8]. Significantly, hemp’s short growing cycle, decreased need for pesticides, and low plant maintenance makes it an ideal candidate for phytoremediation studies [10].

The purpose of this study was to explore the potential of industrial hemp as an eco-friendly way to remediate abandoned mine land soils in Pennsylvania. To do that, physiological parameters such as seed germination, plant height, and days-to-flowering were examined. In addition, uptake of heavy metals such as Arsenic, Lead, Nickel, Mercury, and Cadmium, changes in soil pH, and total cannabinoid content were examined in six different varieties of industrial hemp (Fedora 17, Felina 32, Ferimon, Futura 75, Santhica 27, and USO 31) grown on two contaminated soil types and two commercial soil types both outdoors and in the greenhouse. Additionally, this study presents a detailed review on the possible mechanisms of metal uptake.

## Materials and methods

### Plant material and experimental design

Industrial hemp (*Cannabis sativa* L.) seeds (Fedora 17, Felina 32, Ferimon, Futura 75, Santhica 27, and USO 31) were obtained from the Pennsylvania Department of Agriculture—Bureau of Plant Industry under regulated permits IH-16-P-2017 and IH-17-P-2017 to perform all the experiments. Both fiber and seed/oil varieties of hemp were included in this study. Experiments were set up both outside and within the NIH level II greenhouse of Penn State Harrisburg’s Central Pennsylvania Research and Teaching Laboratory for Biofuels.

Four different soil types, mine land 1 (New Port, PA, USA), mine land 2 (New Port, PA, USA), Miracle-Gro Potting Mix (Marysville, OH, USA), and PRO-MIX HP Mycorrhizae High Porosity Grower Mix (Rivière-du-Loop, Québec, Canada) were used in this study. For every soil type except Miracle-Gro Potting Mix, three individual pots for each of the six hemp varieties were used making it a total of 18 pots per soil type. For the Miracle-Gro Potting Mix, two individual pots for each of the six hemp varieties were used making it a total of 12 pots. This entire set up of 66 pots representing all four soil types was duplicated for both outside and inside the greenhouse making it a total of 132 pots for the entire study.

For each individual pot (22.9 cm diameter x 21.6 cm deep), a total of three seeds were sown. After seed germination data was collected, seedlings were thinned to one plant per pot for the remainder of the study. The greenhouse was maintained at 26°C with a 16-hour light photoperiod at 25–40 μEm-2s^-1^. Relative humidity was maintained at 60 percent. Relevant breeding information for all varieties can be found in Table 1 [11].

**Table 1:** Breeding information on hemp varieties.

Hemp Variety	Genotypic Expression	Recommended Purpose	Vegetative Cycle (days)	Height at Maturity (cm)	Biomass (t/ha)	CBD content (%)	THC content (%)
Fedora 17	Monoecious	Grain/CBD/Fiber	<125	200–250	8–10	1.5–2.0	<0.06
Felina 32	Monoecious	Grain/CBD/Fiber	<135	250–350	8–10	1.5–2.0	<0.12
Ferimon	Monoecious	Grain/Fiber	<125	200–250	<8	1.0–1.5	<0.06
Futura 75	Monoecious	Grain/CBD/Fiber	<145	250–350	10–12	1.5–2.0	<0.12
Santhica 27	Monoecious	Grain/Fiber	<135	200–250	8–10	1.0–1.5	<0.02
USO 31	Monoecious	Grain/Fiber	<125	200–250	<8	0.5–1.0	<0.06

### Seed germination, plant height and days to flowering

Seed germination was recorded after one week of planting. Plant height was recorded weekly over the course of nine weeks and days to flowering was monitored as indicated by the formation of the first flower buds.

### Sample collection and determination of heavy metal content in the soil and leaves

Soil samples (Miracle-Gro and mine land 1 soil from outdoors and in the greenhouse) and the leaves closest to the soil from each individual plant within each treatment were collected for heavy metal analysis in triplicates. The Department of Environmental Protection (DEP) Chapter 271 General Soil Permit test was performed on the initial soil samples from these two soil types by the Agricultural Analytical Services Laboratory (University Park, PA). The test performed screened for Arsenic, Cadmium, Copper, Lead, Mercury, Molybdenum, Nickel, Selenium, Zinc, and polychlorinated biphenyls to satisfy the requirements of DEP Chapter 271, General Permit for Land Application of Sewage Sludge. The test also measured the soil pH. Three biological replicates per treatment were combined and digested [12] using the Environmental Express AutoBlock Plus for acid digestion of soil. Samples were analyzed using the Varian Inc. 720/730-ES Inductively Coupled Plasma-Optical Emission Spectrometer (ICP-OES) with axial plasma following EPA (Environmental Protection Agency) method 6010 for Arsenic, Lead, Nickel, Mercury, and Cadmium.

Leaf samples were harvested in three biological replicates per treatment, flash frozen in liquid nitrogen, stored in the -80°C freezer [13], and prepared for heavy metal analysis using (EPA method 3051) Microwave Assisted Acid Digestion at the ALS Laboratory (Middletown, PA). Samples were then analyzed for Arsenic, Lead, Nickel, Mercury, and Cadmium using Manual Cold-Vapor Technique and Inductively Coupled Plasma-Mass Spectrometry (EPA methods 6020A and 7471B).

### Quantitative analysis of cannabinoid potency

Floral buds from 70-day-old plants from variety Fedora 17 were analyzed for cannabinoid potency by measuring total cannabidiol (CBD) and Δ9- tetrahydrocannabinol (THC) at the Analytical 360 Laboratory (Seattle, Washington). A total quantity of 500 mg of floral bud tissue from the Fedora 17 variety was harvested from indoor and outdoor plants grown on mine land 1 or Miracle-Gro soils. Methanol was used to extract the secondary metabolite compounds. The plant material was then analyzed using the Hewlett-Packard 1050 High Performance Liquid Chromatography (HPLC) equipment based on the De Backer method [14].

### Quantitative Real-Time PCR

Total RNA was extracted from 500 mg of leaf tissue using the Spectrum^TM^ Plant Total RNA Kit (Sigma, St. Louis, MO, USA) and cDNA was synthesized using the High-Capacity cDNA Archive Kit (Life Technologies, Frederick, MD, USA) following the manufacturer’s instructions. Optical densities of total RNA were measured using a Nanovue Plus. A 260/280 nm ratio of 1.8–2.0 for RNA was used to define sample purity. Quantitative RT-PCR reactions were performed using 150 ng of cDNA in a 96 well plate using 5 μL of 2X SYBR Select Master Mix (Life Technologies, Frederick, MD, USA), 1 μL of diluted cDNA reaction mixture, and 4 μL each of 400 nM concentration of forward and reverse primers. The GeneScript Primer Design Program (https://www.genscript.com/sslbin/app/primer) was used to design the primers (Sigma, St. Louis, MO, USA). The accession numbers for the genes and transporters tested, as well as the forward (Fw) and reverse (Rv) primer sequences for the quantitative RT-PCR reaction are found in Table 2 and Table 3.

**Table 2:** Gene accession numbers.

Cs Tetraketide synthase	AB164375
Cs Olivetolic acid cyclase	JN679224
Cs Aromatic prenyltransferase	HK205031
Cs Tetrahydrocannabinolic acid synthase gene	AB057805
Cs Cannabidiolic acid synthase gene	AB292682
Cs Phosphate transporter 1;1 gene	PK29753
Cs Phosphate transporter 1;4 gene	PK29023
Cs Heavy metal ATPase gene	PK06569
Cs Cation exchanger 2 gene	PK18825

**Table 3:** Forward (Fw) and reverse (Rv) primer sequences.

TKS Fw	5’- CACGAGATGCAAACTCTGGA-3’
TKS Rv	5’- TGGTTGATGCGCTAGTGAAG -3’
OAC Fw	5’- CACAGAAGCCCAAAAGGAAG-3’
OAC Rv	5’- TCTTTCATGGCTGGGATGAT-3’
APT Fw	5’- AACTTTGGGAAGGCATGTTG-3’
APT Rv	5’- CCACAAGCGCATGAAGTAAA-3’
CBDAS Fw	5’- GATCCGCTGGGCAGAACGGT-3’
CBDAS Rv	5’- ATGAGGGAATGGAATTGCTG-3’
THCAS Fw	5’- GATCAGCTGGGAAGAAGACG-3’
THCAS Rv	5’- ATGAGGGAATGGAATTGCTG-3’
F-Box Fw	5’- TATCGGCGGAGAGATTTGAG-3’
F-box Rv	5’- TAAGCCCTTCCCTTGATTCC-3’
PHT1;1–2 Fw	5’- CTCGGGTTTGTCCTTTGGTA-3’
PHT1;1–2 Rv	5’-GGCTATGAAAGCTCCACGAG-3’
PHT1;4–2 Fw	5’- GATGTGGCCAAAACACAATG-3’
PHT1;4–2 Rv	5’-GCTTCTCAGCACCATCAACA-3’
HMA3-2 Fw	5’- CGATGCACGATTAGAAGCAA-3’
HMA3-2 Rv	5’- CCCGAGAGCTAACCATTCAA-3’

The reactions were performed using the following conditions: 10 minutes at 95°C, and 40 cycles of the one step cycling of 10 seconds at 95°C, 15 seconds at the standardized annealing temperature for each specific primer (temperatures ranging from 48–95°C), and 1 minute at 60°C. The qRT-PCR reaction ended with a final cycle of 15 seconds at 95°C, 15 seconds at 60°C, and 15 seconds at 95°C. Each qRT-PCR reaction was performed in triplicates on individual biological samples. Relative quantification of specific cDNA levels was performed using the cycle threshold 2^−ΔΔCT^ method (Software IQ5 2.0; [15]). Expression values were normalized using the housekeeping gene F-Box standard (Sigma, St. Louis, MO, USA).

### Data collection and statistical analysis

Statistical Analysis was completed using a 2-Way ANOVA using Tukey’s Multiple Comparison Test [16].

## Results and discussion

### Seed germination

The events associated with seed germination reflects the uptake of water from the soil leading to the emergence of the radicle and visible extension of the shoot [17]. Seed germination has been shown to be influenced by acidic pH due to rapid uptake of nutrients by the roots during the phase of seedling growth [18]. Seed germination data was collected one-week post planting as shown in Table 4.

**Table 4:** Average seed germination percentages (± standard deviation) after one week.

Variety Name	Outdoor				Greenhouse			
	Miracle-Gro^a^	Pro-Mix^a^^c^	mine land 1^b^	Mine land 2^b^	Miracle-Gro^a^	Pro-Mix^a^	mine land 1^b^^c^	Mine land 2^b^
Fedora 17^d^	100.00±0.00	100.00±0.00	33.33±47.14	22.22±41.57	83.33±37.27	88.89±31.43	100.00±0.00	11.11±31.43
Felina 32^d^	66.67±47.14	66.67±47.14	11.11±31.43	33.33±47.14	100.00±0.00	77.78±41.57	22.22±41.57	33.33±47.14
Ferimon^d^	100.00±0.00	44.44±49.69	44.44±49.69	22.22±41.57	100.00±0.00	88.89±31.43	11.11±31.43	22.22±41.57
Futura 75^d^	83.33±37.27	77.78±41.57	22.22±41.57	33.33±47.14	66.67±47.14	66.67±47.14	77.78±41.57	44.44±49.69
Santhica 27^d^	50.00±50.00	88.89±31.43	66.67±47.14	0.00±0.00	83.33±37.27	44.44±49.69	33.33±47.14	11.11±31.43
USO 31^d^	66.67±47.14	0.00±0.00	11.11±31.43	0.00±0.00	16.67± 37.27	44.44±49.69	0.00±0.00	0.00±0.00

^abcd^Letters not shared indicate a p< 0.5.

Statistical analyses using a 2-Way ANOVA test and a Tukey’s Multiple Comparison Test showed no significant difference in seed germination among hemp varieties, in the experimental control Miracle-Gro, and Pro-Mix, in both outdoor and in the greenhouse conditions. A notable difference was observed between both mine land soils and Miracle-Gro and Pro-Mix. Germination on average was higher in Miracle-Gro and Pro-Mix compared to mine land soils. Two weeks after seed germination data was taken, all germinating plantlets in mine land 2 soil died possibly due to a low pH of 3.8 and a compact rock-like soil consistency. The USO 31 plantlets growing outdoors in mine land 1 soil died after germination and USO 31 seeds in the greenhouse germinated after one week. Following a thorough investigative literature search, no similar experiment has been executed with these six varieties of hemp, however, Linger et al. [8] reported no differences in germination percentage under exposure to Cadmium using the USO 31 variety. A recent study completed on the effect of landfill leachate on germination showed that leachate was toxic to the three tested hemp cultivars, however, the leachate was at a more basic pH than the mine land soils in this experiment [19].

### Plant height

The presence of heavy metals in the soil impacts physiological and biochemical processes, thus influencing the overall uptake of nutrients, altering plant growth significantly [20], [21]. Plant height is a critical factor for fiber yield, allowing more surface area for metals to accumulate for phytoremediation. Plant height data was collected weekly for a total period of nine weeks. Average plant height at nine weeks is shown in Table 5.

**Table 5:** Average plant height (centimeters±standard deviation) at nine weeks.

Variety	Outdoor			Greenhouse		
	Miracle-Gro^a^^b^	Pro-Mix^d^	mine land 1^d^	Miracle-Gro^a^	Pro-Mix^b^^c^	mine land 1^c^
Fedora 17^e^	89.00±3.00	24.33±2.05	51.00±13.00	105.75±1.25	61.00±6.68	68.67±23.33
Felina 32^e^	94.00±5.00	33.00±4.55	49.33±5.31	141.00±4.00	53.33±4.71	48.33±18.80
Ferimon^e^	94.00±3.00	16.33±13.27	39.50±30.50	84.00±20.00	75.67±21.31	58.00±26.73
Futura 75^e^	78.5±3.50	31.17±4.19	44.00±0.00	96.00±14.00	79.67±21.31	71.00±7.79
Santhica 27^e^	59.50±17.50	23.17±6.96	58.33±14.82	99.50±7.50	60.33±30.44	64.00±3.00
USO 31^e^	105.75±1.25	16.67±5.19	0.00±0.00	107.00±17.00	76.67±13.96	52.00±0.00

^abcde^Letters not shared indicate a p<0.5

There were no statistical differences in plant height between mine land 1 or Pro-Mix soils that were set up outdoors or indoors. Despite the high level of metal contaminants in the mine land 1 soil, the plant height was comparable in both experimental conditions. When comparing Miracle-Gro and mine land 1 soil, there is a significant difference in plant height. In addition, when comparing the Miracle-Gro and Pro-Mix soils, there was a significant difference in plant height in the outdoor experimental condition. It could be concluded that the mycorrhizae in Pro-Mix had a significant effect on industrial hemp plant growth when not grown on contaminated soil. Mycorrhizae has been shown to positively influence height in other plants [22].

Furthermore, there was no significant difference in plant height among varieties on any soil type. This was an important finding. Specifically, any of the six varieties could be recommended to be grown on mine land soil with predictably similar results. The plant height results are in alignment with previous studies with industrial hemp, with no significant changes in plant morphology concomitant with tolerance to the presence of heavy metals [9].

### Days to flowering

**Fig 1:**
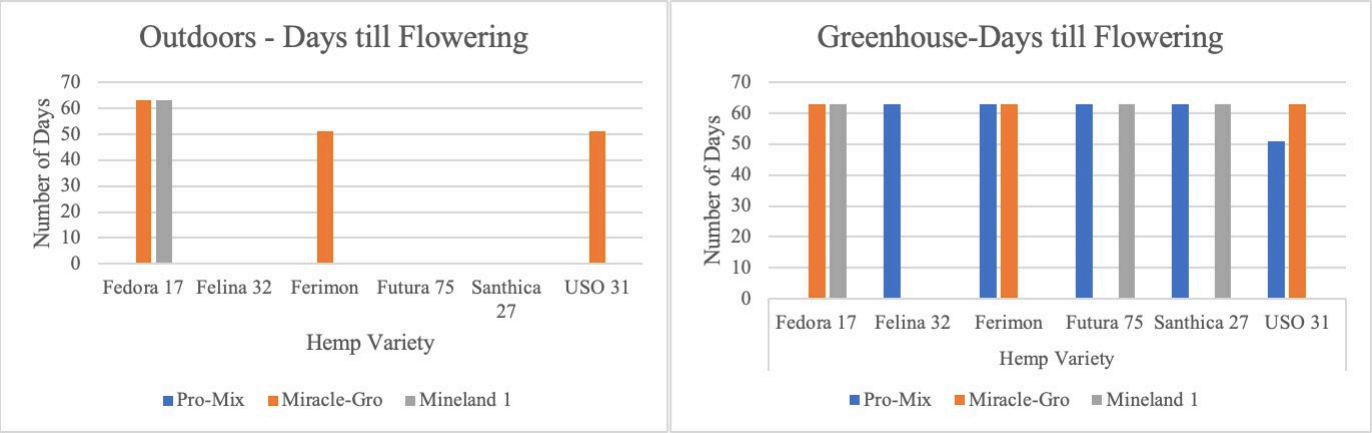
Days to flowering.

Flowering in plants is a physiological phenomenon that is influenced by endogenous plant growth regulators, vernalization, photoperiod, and several other yet-to-be identified factors [23]. Days to flowering is an important factor to cultivar selection as similar flowering times within a variety indicates potential uniformity of the crop in the field [24]. Furthermore, days to flowering is especially a vital factor in industrial hemp as its unique secondary metabolites are produced in the flowers. Additionally, flowering times influence planting dates, as hemp yields maximum fiber growth in the stem a month prior to floral maturity [25]. Days to flowering data was collected for all seed varieties (Fig 1).

Notably, the Fedora 17 variety took a uniform 63 days to flowering independent of the soil medium under both greenhouse and outdoor conditions.

### Soil pH

Soil pH is a factor that can significantly affect the uptake of nutrients from the soil, influencing the bioavailability of metals. Plants can alter the bioavailability through mechanisms of lowering pH and oxygenating the soil [2]. The pH of three soil types was measured initially before the start of the study and after sixty days (Table 6).

**Table 6:** Soil pH in Miracle-Gro, Pro-Mix, and mine land 1 soils initially and after 60 days.

Soil	Initial pH	pH Outdoors after 60 days					pH Greenhouse after 60 days				
		Fedora 17	Felina 32	Ferimon	Futura 75	Santhica 27	Fedora 17	Felina 32	Ferimon	Futura 75	Santhica 27
**Miracle-Gro**	4.6	5.72	5.78	5.66	5.66	5.79	5.88	5.36	5.7	5.44	5.71
**Pro-Mix**	5.8	6.5	6.5	6.5	6.5	6.5	6	6	6	6.5	6
**Mine Land 1**	6.7	7.09	6.68	7.19	7.2	6.54	7.01	6.91	7.21	7.3	7.13

Only five varieties of hemp were included as USO 31 did not grow outdoors. Following 60 days of plant growth, there was an increase in soil pH in the Miracle-Gro and Pro-Mix soils that were hosting all five hemp varieties compared to the initial pH measurements both outdoors and in the greenhouse. There was an increase in soil pH in mine land 1 soil where the Fedora 17, Ferimon, and Futura 75 hemp varieties were growing both outdoors and in the greenhouse. The growth of these varieties caused an alteration in the soil pH indicating their potential for increasing the bioavailability of heavy metals to hemp. Alteration of pH also indicates a possible usage of industrial hemp for phytostabilization, which turns metals in the soil into forms that are less lethal to plants [26], [27].

### Soil analysis for arsenic, lead, nickel, mercury, and cadmium

Heavy metals in soil play a pivotal role in influencing a plant’s morphology as well as the plant’s biomass. A previous study [28] on industrial hemp and heavy metal stress showed specific hypomethylation of DNA, indicating the importance of analyzing metal contents of soil. DNA methylation in plants can have significant impacts on gene regulation and in turn impact the overall plant uptake physiology [29]. In addition, different heavy metals in the soil can interact and impact the plant together through specialized translocation pathways [30]. Initial soil analysis of Arsenic, Lead, Nickel, Mercury, and Cadmium metals were measured at sixty days (Table 7) for mine land 1 and Miracle-Gro soils.

**Table 7:** Heavy metal content (mg/kg dry weight) initially and at 60 days in Mine land 1 and Miracle-Gro soils.

Mine Land 1											
Elements	Initial	Outdoors					Greenhouse				
		Fedora 17	Felina 32	Ferimon	Futura 75	Santhica 27	Fedora 17	Felina 32	Ferimon	Futura 75	Santhica 27
As	8.65	9.6	8.98	8.97	8.77	9.09	8.19	8.91	7.89	8.11	8.41
Cd	0.34	0.39	0.39	0.34	0.35	0.38	0.28	0.36	0.37	0.32	0.32
Ni	12.83	13.96	13.77	13.64	13.51	13.34	12.88	13.47	13.31	13.27	13.26
Pb	13.74	15.94	15.22	14.07	14.4	16.4	14.01	15.45	13.74	13.51	13.78
Hg	0.043	0.046	0.056	0.046	0.045	0.046	0.048	0.046	0.046	0.045	0.049
Miracle-Gro											
Elements	Initial	Outdoors					Greenhouse				
		Fedora 17	Felina 32	Ferimon	Futura 75	Santhica 27	Fedora 17	Felina 32	Ferimon	Futura 75	Santhica 27
As	1.75	1.04	1.21	1.29	1.25	1.15	1.49	1.86	1.53	1.39	1.25
Cd	< .25	< .25	< .25	< .25	< .25	< .25	< .25	< .25	< .25	< .25	< .25
Ni	8.62	4.08	3.81	5.39	4.82	4.93	7.27	8.14	9.67	6.78	6.07
Pb	4.18	3.69	3.96	4.52	3.95	4.13	5	5.47	4.83	4.66	4.82
Hg	0.073	0.057	< .034	< .038	0.047	0.042	0.037	0.037	0.041	0.034	0.035

Soil analysis revealed that mine land 1 soil had significantly higher heavy metal contaminant levels when compared to the Miracle-Gro control soil compared before and after the plants were grown for 60 days.

### Leaf analysis for arsenic, lead, nickel, mercury, and cadmium in Felina 32

The fate of metal uptake because of ion exchange and transport through the roots leads to heavy metal accumulation in the vegetative tissues [30]. Linger et al. [7] reported the leaves to contain the highest concentrations of Lead, Nickel, and Cadmium in industrial hemp indicating the movement of heavy metals through the xylem sap. *Brassica juncea*, a commonly used plant for phytoremediation, also indicated the highest concentration of metals was in the leaves [30]. Thus, the lowermost leaves of the Felina 32 variety from 60-day old plants were collected for Arsenic, Lead, Nickel, Mercury, and Cadmium metal analysis (Table 8).

**Table 8:** Metal analysis of Felina 32 leaf (mg/kg dry weight) after 60 days.

Elements	RDL	Miracle-Gro Greenhouse	Mine Land 1 Greenhouse	Mine Land 1 Outdoors
As	0.29	ND	ND	ND
Cd	0.096	ND	0.38	0.16
Pb	0.19	0.19	ND	0.3
Hg	0.05	ND	ND	ND
Ni	0.48	ND	0.59	1.5

Note: ND = Not Detected, RDL = Real Detection Limit

The Nickel concentration was 2.5 times more in the Felina 32 variety’s leaves collected from plants grown on mine land 1 soil outdoors compared to leaves collected from plants grown on mine land 1 soil indoors. This could possibly be correlated to the release of bound Nickel into a soluble form. Cadmium is detected in the leaf tissue in mine land soils both indoors and outdoors and not in Miracle-Gro indoors.

### Mechanism of heavy metal uptake by plant

In the present study, the capacity of Arsenic, Lead, Nickel, Mercury, and Cadmium uptake by the Felina 32 variety of plants after sixty days of growth was investigated.

The plants of the Felina 32 variety were able to uptake certain heavy metals in different capacities. Results indicate Ni to be most absorbed in the highest quantity (1.5 mg/kg dry weight) while As and Hg were not detectable. Findings indicate the Felina 32’s variety’s metal uptake capacity from highest to lowest capacity to be Ni > Cd > Pb > As = Hg. A comprehensive investigation is needed to understand the underlying heavy metal uptake and transport mechanisms and the role they play in the phytoremediation process.

#### Lead properties and uptake mechanism

Results of this study shows that the Felina 32 variety exhibited a higher uptake of Pb in the mine land 1 soil grown outdoors (0.3 mg/kg dry weight) than the Miracle-Gro greenhouse-grown soil (0.19 mg/kg dry weight). The results suggest that mechanisms in place for Pb uptake in the Felina 32 variety, may have increased efficacy in its function at lower temperatures (approximately 16.7°C).

#### Nickel properties and uptake mechanism

In the present study, Ni exhibited its highest uptake in mine land 1 soil compared to As, Cd, Pb, and Hg. The Felina 32 variety expressed higher Ni uptake when grown outdoors (1.5 mg/kg dry weight) as compared to in the greenhouse (0.59 mg/kg dry weight). Maximum uptake functions were best at 16.7°C, the average outdoor temperature during its time of growth. This study findings suggest that industrial hemp is a great candidate for Ni phytoremediation.

#### Mercury properties and uptake mechanism

In the present study, Hg uptake, similarly to As, was not detected in the Felina 32 variety. The findings suggest that industrial hemps’ inability to uptake Hg may be due to Mercury’s highly variable charges, due to its unique electron configuration, playing perhaps a key role in its uptake through plant root cells [6], [30].

#### Cadmium properties and uptake mechanism

Results of this study show higher amounts of Cd uptake in the Felina 32 variety (mine land 1) grown in the green house (0.38 mg/ kg dry weight) than when grown outdoors (0.16 mg/ kg dry weight). Results suggest that industrial hemp, specifically the Felina 32 variety, may be utilizing metal transporters similar to *Arabidopsis*, which seem to have increased efficacy at 26°C, the temperature at which the greenhouse was maintained. Industrial hemp displayed high tolerance to Cd making it a promising candidate for phytoremediation and calls for further research on its capacity to uptake and remediate Cadmium.

### Gene Expression analysis of heavy metal transporters

**Fig 2:**
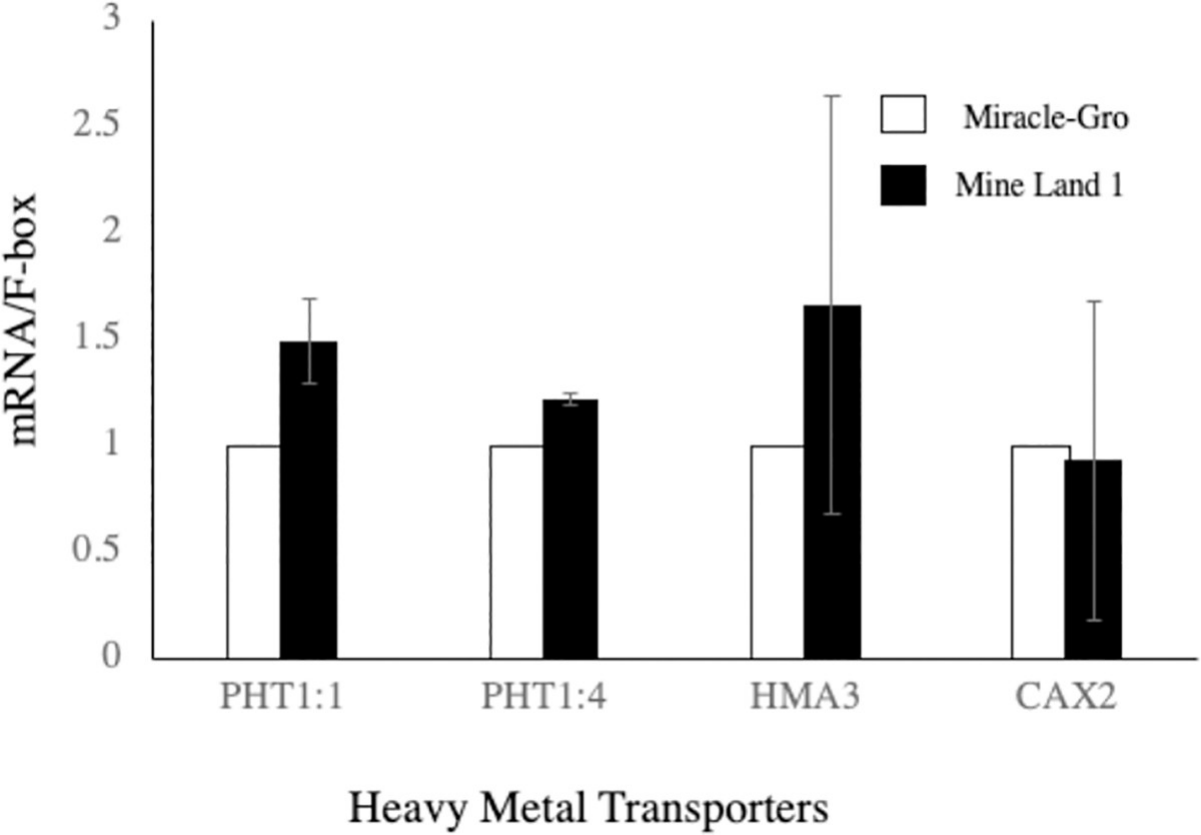
Gene expression analysis of heavy metal transporter genes.

Metal cation uptake is regulated by 1) metal uptake through root, 2) loading into the xylem, 3) translocation, chelation and sequestration, and 4) phloem translocation/movement. In the first step of symplastic pathway, some metal cations are transported across the root membrane whereas others are not incorporated into root cellular in apoplastic pathway [31]. For influx to the symplasm, a lot of proteins such as ZRT- and IRT-like proteins, stripe-Like (YSL) transporters, natural resistance-associated macrophage proteins (NRAMP), channels and other genes related to Cd-uptake/restriction play critical roles [32]. In both symplastic and apoplastic pathways, loading of metal cations into the root xylem is critical for them and redistribution of metal cations across the different plant tissues is dependent on this step. This loading is mediated by HMA2 and/or HMA4 [33]. Within plant cells, metal cations are bound to chelatins, chelatin synthesis pathway genes, and their transporters also play critical roles for uptake of metal cations [34]. On the other hand, metal cations are also transported through phloem, and xylem-phloem transfer is indicated to be a key process for the transfer to plant leaves and grains [35]. Thus, xylem-phloem transfer transporters are involved in heavy metal uptake in plant. In this study, gene expression of representative transporter genes was measured such as Phosphate transporter (Pht) 1;1, Pht1;4, Heavy metal ATPase 3 (HMA3), Cation exchanger 2 (CAX2). There was no difference in gene expression of four genes between mine land and Miracle-gro (Fig 2).

The mechanism underlying heavy metal transport and accumulation is a dynamic process and more than 100 proteins are indicated to be involved in this process. Transcriptomic and proteomic analyses are the next step for a deeper understanding of hemp behavior under heavy metal stress from a molecular level.

### Gene expression of secondary metabolites

The presence of heavy metals in the soil influences the mechanism of uptake of secondary metabolites in plants, because of oxidative stress, and eventually may be causing lipid peroxidation to stimulate the production of bio-active compounds [36], [37]. Industrial hemp’s secondary metabolites, such as cannabinoids and terpenoids, are found in the glandular trichomes of the cannabis plant [38]. Total CBD and total THC was analyzed from the floral buds of the Fedora 17 variety grown on the field, mine land 1, and Miracle-Gro soils (Table 9), and was then compared to published values in Table 9.

**Table 9:** Cannabinoid contents (%) in floral buds of Fedora 17 in field conditions, Miracle-Gro, and mine land 1.

Cannabinoids	Konopko*	Field	Miracle-Gro		Mine Land 1	
			Outdoor	Greenhouse	Outdoor	Greenhouse
Δ9-THC-A	-	< .01	0.06	0.42	< .01	0.12
Δ9-THC	-	< .01	< .01	0.03	< .01	0.02
Δ9-THCV	-	< .01	< .01	< .01	< .01	< .01
Δ8-THC	-	< .01	< .01	< .01	< .01	< .01
CBN	-	< .01	< .01	< .01	< .01	0.06
THC-TOTAL	< .06	< .01	0.07	0.4^&^	< .01	0.12
CBD-A	-	0.26	1.22	1.8	2.47	2.89
CBD	-	< .01	0.01	0.02	< .01	0.05
CBDV-A	-	< .01	< .01	< .01	< .01	< .01
CBDV	-	< .01	< .01	< .01	< .01	< .01
CBD-TOTAL	1.5–2	0.23	1.08	1.6	2.16	2.58

* theoretical values from Table 1

- information not available

^&^any material that increased the THC levels due to heavy metal stress was destroyed

Total THC content in the floral buds collected from plants grown in Miracle-Gro soil outdoors or in the greenhouse was higher than the floral buds collected from plants grown in mine land soil outdoors or in the greenhouse and field conditions. Total THC levels in mine land 1 soil with the Fedora 17 variety remain under the European Economic Community (EEC) legal threshold limit of 0.3%, whereas the Fedora 17 variety in Miracle-Grow indoors is over the legal limit for industrial hemp. Total CBD content in the floral buds grown in mine land 1 soil in both outdoors and in the greenhouse was higher than the floral buds grown in Miracle-Gro in both environmental parameters and in the field, which can be concluded due to the heavy metal stress.

**Fig 3:**
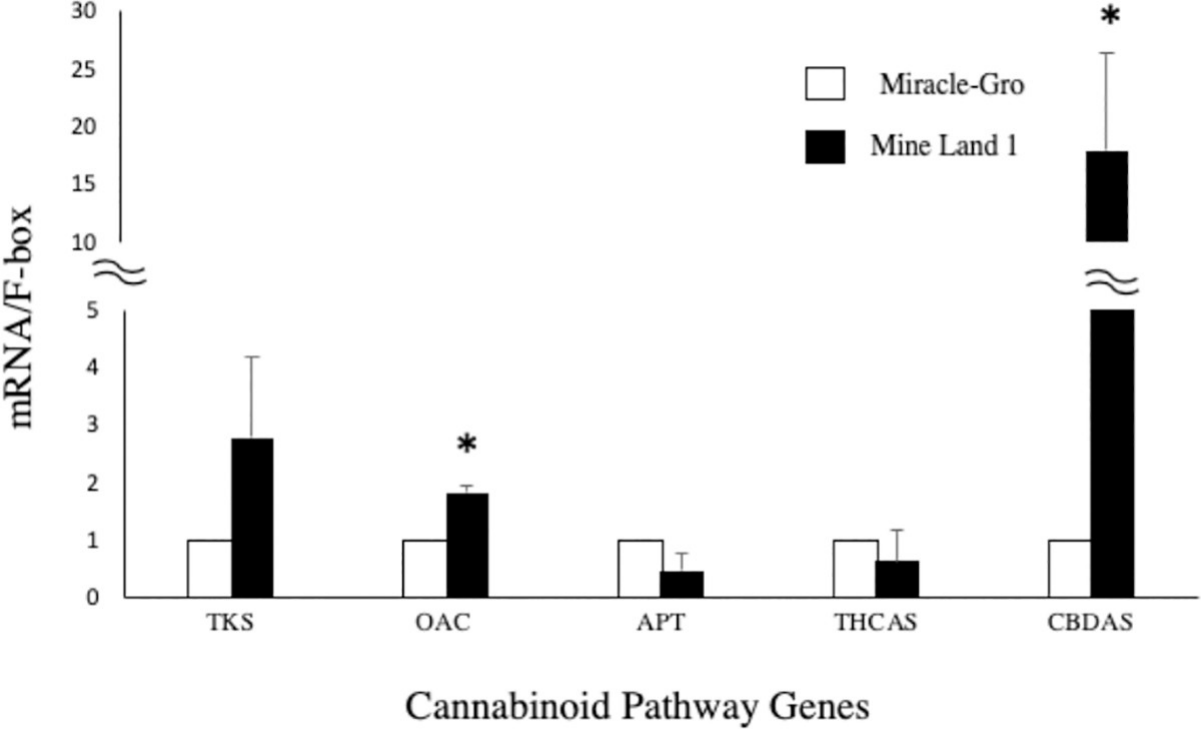
The gene expression of Cannabinoid pathway genes in mine land and Miracle-Gro soil. Hemp plants grown under Miracle-gro soil were used for normalization. Bars ±standard error of the mean. Asterisk means significant difference in statistical analysis (P<0.05, n = 3).

To estimate the alteration of gene expression of hemp in outdoor mine land conditions, a qRT-PCR was performed on the Ferimon variety. Cannabinoid pathway starts with the synthesis of olivetolic acid by tetraketide synthase and olivetolic acid cyclase [39]. Afterwards, olivetolic acid is converted into Cannabigerolic acid (CBGA) by CBGA synthase. Finally, THCA and CBDA are synthesized from CBGA by THCA synthase (THCAS) and CBDA synthase (CBDAS), respectively [40]. In this study, we measured gene expression of five cannabinoid pathway genes above. Notably, Cannabidiolic acid synthase (CBDAS) was expressed 18 times higher in the mine land soil, however there was no significant difference between mine land 1 and Miracle-Gro soil in the tetrahydrocannabinolic acid synthase (THCAS) expression (Fig 3).

Interestingly, among the other three upstream genes, olivetolic acid cyclase gene expression was also enhanced in mine land 1. In summary, hemp increased total CBD content under high heavy metal conditions and was a result of enhancement of CBDAS and OAC gene expression.

This is the first study to demonstrate that gene expression of CBDAS can be remarkably influenced by mine land soil conditions which agrees with an increase in secondary metabolite production in *Camellia sinensis* [41]. A recent study showed that hemp could improve phenolic production when used for phytoremediation of heavy metals [42]. These results provide promising insight into the metabolic networking mechanism and the signaling cross-talk that could occur during the biosynthesis of CBD and THC molecules, just two of the 100 known cannabinoids of industrial hemp [43].
